# tsRNA's Biological Function and its Potential Application in Disease Diagnosis and Prognosis

**DOI:** 10.7150/jca.135857

**Published:** 2026-07-14

**Authors:** Congcong Tang, Qi Tang, Siyuan Tang, Faqing Tang

**Affiliations:** 1 Hunan Key Laboratory of Oncotarget Gene, Hunan Cancer Hospital, and The Affiliated Cancer Hospital of Xiangya School of Medicine, Central South University, Changsha 410013, China.; 2 Xiangya School of Nursing, Central South University, Changsha, China.

**Keywords:** tsRNA, regulatory mechanism, biomarker, non-tumor disease, tumor

## Abstract

Under physiological conditions, tsRNAs regulate mRNA transcription, reverse transcription, and protein translation to mediate cell apoptosis, cell cycle, and epigenetic regulation. Pathogenic tsRNAs are disease-specific or disease-related tsRNAs that are highly express in various diseases and may serve as diagnostic or potential diagnostic biomarkers. Specifically expressed tsRNAs in tumors were screened and obtained from the tumors. Although the mechanisms by which these oncogenic tsRNAs contribute to tumor development remain unclear, they have been shown to be applicable to tumor diagnosis and therapeutic prognosis. This article briefly summarizes pathogenic tsRNAs involved in various diseases and their biological functions. Oncogenic tsRNAs in tumors and their clinical applications have been elaborated upon. The molecular mechanisms of pathological tsRNAs in both general diseases and tumors need to be further investigated in future.

## Introduction

tRNA is a fundamental component of the translation mechanism that delivers amino acids to ribosomes and translates genetic information into corresponding peptide chains. It is the most abundant of all non-coding RNA small molecules, accounting for 4-10% of all cellular RNA 1. Aminoacyl-tRNA is activated, accurately transferring amino acids to the peptide chain. tsRNAs are small RNAs derived from tRNAs and also known as tRNA-derived fragments (tRF), tRNA-derived stress-induced RNAs (tiRNAs), and semi-tRNA. tsRNAs are a recently discovered class of small non coding RNAs, produced by the cleavage of mature tRNA or tRNA precursors by enzymes, such as angiogenin (ANG), Dicer, RNase Z, and RNase P 2. tRNA genes are transcribed into precursor tRNAs (pre-tRNAs) through RNA polymerase III (RNA Pol III), each of which has a 5'leading sequence and a 3' tail region 3. Usually, RNase P removes the leading segment to the 5'end 4, and RNase Z removes the trailing segment at the 3' end 5; nucleotide transferase adds the “CCA” sequence to the 3 'end 6; subsequently the sequence is folded into the secondary clover structure of mature tRNA by post transcriptional modification. The structure of tRNA clover consists of a dihydrouracil ring (D ring), dihydrouracil arm, anticodon ring, anticodon arm, variable ring, pseudouracil ring (T ψ C ring or T ring), pseudouracil arm, and amino acid arm 3. tsRNAs exert biological functions through various mechanisms, such as interacting with proteins or mRNA, inhibiting translation, and regulating gene expression, cell cycle, chromatin, and epigenetic modifications 7,8. Recently, some disease-specific and disease-related tsRNAs have been discovered 9-14, and are known as pathogenic tsRNA. They have been verified to play critical biological functions in the occurrence and development of diseases 15,16, and some tumor-specific and tumor-related tsRNAs have been screened and applied in tumor diagnosis and therapy 17-22. This article systematically expounds tsRNA formation mechanisms and biofuncation. We also briefly summarized the tsRNAs applications of tsRNA in disease diagnosis. In particular, the diagnostic application and therapeutic prognosis of patients with tumors are importantly elaborated.

## 1. Formation mechanisms of tsRNA and biological functions

tsRNAs include i-tRF, tRF-1, tRF-2, tRF-3, tRF-5, tiRNA, sitRNA-5, and sitRNA-3 23-25. tRFs and tiRNAs are a class of small non-coding RNAs produced by site-specific cleavage of mature or precursor tRNAs, but their nomenclature remains inconsistent. Collectively, termed tRNA-derived small RNAs or tRNA-derived RNAs, these molecules are known by multiple interchangeable names, including tRFs, tiRNAs, tRNA halves, SHOT-RNAs, and tsncRNAs. The most widely adopted classification divides them into two main groups, longer tiRNAs (29-50 nt) and shorter tRFs (14-30 nt). The former is also called stress-induced tRNA halves, which are generated by angiogenin-mediated cleavage within the anticodon loop into 5′-tiRNA and 3′-tiRNA. The latter is further subdivided according to their origins, tRF-5 from the 5′ end (cleaved at the D-loop), tRF-3 from the 3′ end (cleaved at the T-loop, containing CCA motif), tRF-1 from the 3′ trailer of pre-tRNAs (with a poly-U tail), and internal i-tRFs spanning central regions. In addition, the databases of tRFdb use numerical IDs to name tRF, such as 5001 for tRF-5 and 3001 for tRF-3, while other systems incorporate tRNA isotype, amino acid, anticodon, or genomic position. Some research groups adopt distinct naming conventions based on fragment length (5′ tRF, 3′ tRF, and i-tRF), processing origin (tRF-1, tRF-2, tRF-5a/b/c), or the specific tRNA isodecoder from which they originate, such as 5′ tRFs derived from the 5′ end of mature tRNAs, 3′ tRFs from the 3′ end, and internal tRFs (i-tRFs) from internal regions are commonly described 26,27.

i-tRF, tRF-2, tRF-3, and tRF-5 are derived from mature tRNAs through cleavage by ANG 28 and Dicer 29, or from other tRNAs that RNase cleavages RNA at different sites of RNA. tRF-1s are derived from pre-tRNAs cleaved by RNase Z decomposing. tiRNAs are also classified into two types, 5'tiRNA and 3'tiRNA. They are derived from mature tRNAs that are cleaved from the anticodon loop. A fragment longer than the RNA is called stress-induced sitRNA-5 or sitRNA-3 30. tRF-5 is usually present in the nucleus 31, whereas tRF-1 and tRF-3 are mainly present in the cytoplasm 32,33. Methylation 34 is the most common modification of tsRNA, and some tRNAs that contain 5'-terminal oligoguanine can regulate the expression of 5'- tRFs through pseudouridylation 35. Nucleoside modification 36 also plays important roles in tsRNA biology. The main biological functions of tsRNA are described as follows.

### 1.1 tsRNA regulates mRNA transcription

Recent studies have shown that tsRNAs recognize target mRNAs and bind to them during transcription, when the untranslated region is replaced and the transcriptional stability is decreased, thereby inhibiting mRNA expression 37. Zhang *et al*. discovered a 5'-tRF, which is generated from piRNA (td piR (Glu)) that is derived from 5'-tRNAGlu. 5'-tRF was proved to bind to PIWI-like protein 4 and recruit SET domain bifurcated histone lysine methyltransferase 1, suppressor of variegation 39 homolog 1, and heterochromatin protein 1 β to the cluster of differentiation 1A (CD1A) promoter region, thereby which regulates H3K9 methylation and inhibits human monocyte CD1A transcription 38. Chen *et al*. 39 suggested that sperm tsRNAs interact with promoter regions, but not coding regions, promoting downstream gene expression, and that binding alters the inheritance of metabolic traits and embryonic development. Collectively, stRNAs have respective and intricate molecular mechanisms to participate into cellular biological behavior, stRNAs from different origins regulate gene transcriptions via different mechanism. A group of i-tRFs from tRNAGlu, tRNAAsp, tRNAGly, and tRNATyr bind to the 3 'end of multiple oncogenic transcripts through competing with Y-box binding protein 1 (YB1), which results in a decrease in expression of target oncogenes, thus inhibiting breast cancer metastasis 40. In addition, tsGlnCTG interacts with insulin-like growth factor 2 mRNA-binding protein 1 to inhibit transcript stability and promote stem cell differentiation 41. 3'tsRNAHisGTG and 3'tsRNALeuCAG exert crucial roles in gene expressions, 17nt 3'tsRNAHisGTG and 18nt 3'tsRNALeuCAG induce the cleavage of artificial target mRNA mediated by endogenous argonaute (AGO)2 42. AGO family members are important tsRNA targets. Kumar P *et al*. found that tsRNAs associated with AGOs regulate the gene silencing 31, and further discovered that type I tsRNAs interact more easily with AGO1, AGO3, and AGO4 than with AGO2, whereas type II tsRNAs have almost no interaction with AGO protein. Maute *et al*. 43 evaluated the interaction of CU1276tsRNA (3'tsRNAGlyGCC) with AGO1-4 proteins through immunoprecipitation experiments and found that these interactions repress mRNA transcripts in a sequence-specific manner. The relationship between tsRNAs and AGO protein was verified in fruit flies, and the results showed that the interaction of tsRNAs with AGO1 or AGO2 changes with age 44. The downstream molecules of tsRNAs have some specificity, various AGO-interacting tsRNAs interact with different AGO family members, tsRNA function is determined to some extent by its downstream AGO members.

### 1.2 tsRNA regulates protein translation

tRFs and tiRNA may inhibit protein translation by disrupting ribosome formation and prolonging protein activity 45. Under specific situation, halophilic archaeon *Haloferax volcanii* from tsRNAs produced by tRNAVal5 fragments inhibited peptidyl transferase activity and weakened translation by binding to small nucleosome subunits 46. Arabidopsis can bind to multiple ribosomes to generate tRFs that inhibit plant translation 47. Moreover, 5'-iRNAAla and 5'-iRNACys replace the cap end of the complex eukaryotic initiation factor gamma 4F (eIF4G/A/F) by binding to the translation inhibitory factor YB-1 to induce the assembly of stress granules, thus inhibiting translation initiation 48. Subsequently, G-quadruplex 4 tiRNA was shown to bind directly to the HEAT1 domain of eIF4G, resulting in impaired 40S ribosome binding and translation silencing 49. Another report proved that deletion of cytosin-5 RNA methyltransferase NSun2 leads to an accumulation of 5'- tRFs and a decrease in protein translation rate 50. Pseudouridylate synthase 7 mediated tRF-5 pseudouridylation can inhibit stem cell translation 35. tsRNA5GluCTC mediated by respiratory syncytial virus (RSV) promotes RSV replication, which reduces RSV defense genes expression and tsRNA5GluCTC interaction with RSV proteins 51. The silencing complex formed through tRF-3 binding to Ago3 and Ago4 directly binds to the mRNA of the target gene, ultimately inhibiting its translation of target gene. It was found that 5'-tRFGln19 interacts with triple synthase complex, promoting ribosome binding to poly (A) tail and translation elongation 52.

### 1.3 tsRNA mediates reverse transcription

Some tsRNAs regulate reverse transcription, such as the tRFs originating from the front tail region of tRNA, which regulates viral gene expression by isolating La/SSB from the cytoplasm 53. tRF mediated by tRNAGlyGCC inhibits nearly 70 endogenous reverse transcription factor MERVL related genes transcription 56. RSV infection induces tsRNA5GlyCTC, tsRNA5GlyGCC, tsRNAF5LysCTT, and tsRNA5CysGCA 51,57, tsRNA5GlyCTC mediates RSV replication by inhibiting apolipoprotein E receptor 2 gene transcription 58. tsRNAs targeting the primer binding sites (PBS) of tRNA mediate the inhibition of gene transcription. Moreover, 18 nt 3'-tRF from dsRNA inhibited HIV-1 reverse transcription through targeting PBS, suppressing HIV-1 proliferation 54. Conversely, tRF-3019 binds to PBS as a primer for the human T-cell leukemia virus type 1 reverse transcriptase, enhancing viral infection 55.

### 1.4 tsRNA is involved in cell apoptosis and cell cycle regulation

Thyroid papillary carcinoma (PTC) detection found that 33 nt 5'tiRNAGlyGCC is elevated in PTC, and it binds to the U2AF homology motif domain of RNA binding motif protein 17 (RBM17) and promotes RBM17 to translocate into the nucleus. Stable expressed-RBM17 induces alternative splicing of mitogen-activated protein kinase 4 mRNA exon 16, leading to proliferation and migration of PTC cells 59. The immunohistochemistry detection showed that tsRNA-26576 is highly expressed in breast cancer tissue, and the cell experiments have shown that tsRNA-26576 inhibits cell apoptosis and induces the proliferation and migration of MDA-MB-231 cells 60. 3'tsRNAValCAC2 directly binds to partner molecule eukaryotic translation elongation factor 1α1 (EEF1A1) and mediates its transport to the nucleus, thereby promoting the interaction with p53 E3 ubiquitin ligase MDM2. This indicates that 3'tsRNAValCAC2 inhibits gastric cancer cell apoptosis via the MDM2-p53 pathway, resulting in cancer progression 61. In mouse models, knock-down of 3'tsRNALeuCAG induced significant apoptosis in HeLa cells, and decreased cancer xenografts derived from patients 62. In wild-type mouse embryonic fibroblasts (MEF), high osmotic stress induces the mitochondrial release of cytochrome c (Cyt c) and subsequent formation of apoptotic bodies, finally leading to cell apoptosis. Mechanically, stress granules isolate Cyt C in ribonucleoprotein complexes by inducing 5'- and 3'- tiRNAs, thereby protecting MEF or primary neurons from osmotic stress, inducing apoptosis 63. tRF-1001 is a type of tsRNA-1 derived from the 3'end of tRNASer precursor, it is essential for tumor cell growth 64. Knock-down of tRF-1001 causes cells to remain in the G2 phase by inhibiting DNA biosynthesis, which interferes with cell proliferation 65. Some tRFs and tiRNAs bind to cytochrome C, inhibiting the interaction of cytochrome C with Apaf-1 to block the activation of caspase-9 and restrain apoptotic body formation, thus suppressing apoptosis 63,66. In addition, tRNA methyltransferase 10A mediated by 5'tRNAGln can induce pancreatic beta cell death 67.

### 1.5 tsRNAs in the epigenetic inheritance of acquired traits

Some tRFs from the sperm or epigenetic factors induce the expression of certain offspring genes, causing metabolic disorders. tsRNAs have been reported to act as epigenetic regulators that affect offspring metabolism. Male mice fed a high-fat diet (HFD) developed insulin resistance and impaired glucose tolerance, which occurred 7 weeks after birth and became severe at 15 weeks. This finding was verified to be caused by 30nt-34nt tRFs 68. The high-fat diet of parental mice altered the expression profile of specific tsRNAs (e.g., 5'-tRFGlyGCC) in sperm, and the injection of these tsRNAs into normally fertilized eggs resulted in similar metabolic abnormalities (e.g., insulin resistance) in offspring. This is direct evidence that environmental factors (diet) can pass on acquired metabolic traits to offspring by altering tsRNA profiles in reproductive cells without altering DNA sequences. In addition, tRFGluTTC is considered a novel epigenetic regulator that promotes fat production 69. One finding demonstrates that tRFGly functions as a key regulator of fat accumulation, targeting tRFGly may represent a therapeutic strategy for related metabolic disorders 70. Moreover, this mechanism may be involved the regulation of mitochondrial tsRNA in fat deposition, suggesting that tsRNA may affect the intergenerational inheritance of energy metabolism by regulating mitochondrial function. Another study in the mice fed a low-protein diet confirmed that tsRNAs play critical roles in mammalian sperm maturation and fertilization. A low-fat diet was not associated with tsRNAs in immature sperm from mouse testes but had a significant effect on tsRNAs in mature sperm, particularly with a significant increase in tRNAGlyGCC levels 56. As a RNA modification enzyme, DNMT2 plays a role in this process by changing the modification pattern of tsRNAs 71. The latest research shows that the microRNAs carried by sperm (including tsRNA) not only affect fertilization rate, but also directly determine embryo quality and developmental potential 72. In addition, tRFGluTTC is considered a novel epigenetic regulator that promotes fat production 73.

Biological functions of tsRNAs are summarized in Figure. Briefly, 5'-tRF, i-tRF, and tRF-3 respectively recognize their respective target molecules, inhibiting mRNA expression. 5'-tRF, i-tRF, and tRF-3 inhibit protein translation by disturbing ribosome formation and prolong protein activity. tRF-3 and 5'-tRFs regulate reverse transcription through interaction with reverse transcriptase. i-tRF and tRF-3 regulate cell cycle process via DNA biosynthesis, and participate in cell apoptosis through activating caspase-9 and restraining apoptotic body formation. tRF-3 participates in epigenetic inheritance via affecting the metabolism of offspring. tiRNAs including 5'- and 3'-tiRNAs, induce stress granules to isolate Cyt C in ribonucleoprotein complexes, thereby protecting MEF or primary neurons from osmotic stress.

## 2. tsRNAs from patients with various tumors and clinical significance

### 2.1 tsRNAs are applied in tumor diagnosis

Serum tRFProAGG004 and tRFLeuCAG002 levels can be used as new biomarkers for pancreatic cancer diagnosis, even in the early stage 74. In addition, tsRNAValTAC41 and tsRNAMetCAT37 were validated to have diagnostic value in patients with pancreatic ductal adenocarcinoma 75. Compared with normal controls, tRF-5 derived from tRNAHis is downregulated in chronic lymphocytic leukemia 76. tsRNA-46 and tsRNA-47 are strongly downregulated tsRNAs in chronic lymphocytic leukemia and lung cancer 77. tsRNA-40 is upregulated in colon cancer, ts-66 and ts-86 are significantly dysregulated in breast cancer, and tsRNA-29 is overexpressed in ovarian cancer 77. More interestingly, tRFGlnTTG006 can distinguish hepatocellular carcinoma from healthy individuals with high sensitivity (80.4%) and specificity (79.4%), even in stage I with a sensitivity of 79.0% and specificity of 74.8% 78. In addition, the levels of tRNA-5 in plasma exosomes from patients with liver cancer were significantly higher than those in healthy controls 79. Shen *et al*. suggested that tRF-33-P4R8YP9LON4VDP is a potential biomarker for gastric cancer diagnosis and may be a novel therapeutic target because it inhibits the proliferation of gastric cancer cells 80. This implies that tRF-33-P4R8YP9LON4VDP can be used for the diagnosis and treatment of gastric cancer in future. SVM prediction model for three tsRNAs, tRF-16L85J3KE, tRF-16-PSQP4PE, and tRF-21-RK9P4P9L0, can all predict lung adenocarcinoma 81. tiRNA-1:33-GlyGCC1, tRF-1:32GlyGCC1, and tRF+1:T20SerTGA-1 may participate in the pathophysiological process of muscle-invasive bladder cancer 82. Meanwhile, there are differences in the expression of tRF-1-32-chrm between glioblastoma and low-grade glioma tumor tissues 83. tRFTyr increases lactate accumulation and promotes tumor progression in laryngeal squamous cell carcinoma, which can help develop new diagnostic biomarkers 84. The levels of extracellular vesicles tRFLeuTAA005, tRFAsnGTT010, tRFAlaAGC036, tRFLysCTT049, and tRFTrpCA057 are significantly downregulated in non-small cell lung cancer, and may be promising biomarkers for the diagnosis 85. Wang *et al*. 86 discovered six 5′-end-derived tRFs from tRNA, such as tRFGluCTC003, tRFGlyCCC007, tRFGlyCCC008, tRFLeuCAA003, tRFSerTGA001, and tRFSerTGA002. They may serve as potential novel biomarkers for early-stage breast cancer diagnosis. In addition, tiRNA-1:34GluTTC2 and tRF-60:76ArgACG1M2 may be associated with therapeutic strategies against multiple bone diseases 87. Three tRF subtypes (tRF-3022b, tRF-3030b, and tRF-5008b) displayed elevated expression in colorectal cancer tissues compared to adjacent normal tissues, and their levels were also increased in plasma exosomes from patients when compared to healthy controls 88.

### 2.2 tsRNA predicts therapeutic prognosis for tumor patients

*In situ* hybridization (ISH) scores for tRFProAGG004 and tRFLeuCAG002 may serve as valuable biomarkers for predicting postoperative survival time in patients with pancreatic cancer 74. Balatti *et al*. discovered that ts-36 may play an important role in the malignant transformation of colon cells 76. Additionally, high i-tRFGlyGCC is associated with shorter disease-free survival (DFS) in patients with colorectal cancer, and it is also associated with an increased likelihood of poorer overall survival in this group 89. tRF-21-RKP4P9L0 is linked to the prognosis of lung adenocarcinoma (LUAD) 81. In a therapeutic context, the inhibition of exosomal tRF-16-K8J7K1B enhances sensitivity to tamoxifen in breast cancer, suggesting that tRF-16-K8J7K1B could be a novel therapeutic target for overcoming tamoxifen resistance 90.

## 3. tsRNAs in patients with non-tumor diseases and clinical significance

Some distinct tsRNAs may act as novel diagnostic biomarkers for SLE, osteoporosis, AD, osteoporosis, and OSAHS. tRFHisGTG1 combined with anti-dsDNA was verified to be served as a biomarker for diagnosing systemic lupus erythematosus with an area under the curve of 0.95 (95% CI = 0.92-0.99), a sensitivity of 83.72%, and a specificity of 94.19% 91. Compared with healthy individuals, tsRNA-10277 is significantly downregulated in the plasma exosomes from patients with steroid-induced osteonecrosis 92. In idiopathic pulmonary arterial hypertension (PAH) 35, four upregulated tsRNAs were identified, namely i-tRF-31:54ValCa1, 5'tiRNA-31GluCTC16, tRF-3aAspGTC9, and tRF-3bTyrGTA4, and four downregulated tsRNAs were discovered, namely 5'tiRNA-33LysTTT4, i-tRF-8:32ValAAC2, i-tRF-2:30HisGTG-1, and i-tRF-15:31LysCTT1. Plasma tRF-25, tRF-38, and tRF-18 exosomes may serve as diagnostic biomarkers for the detection of osteoporosis 93. La Ferlita *et al*. reported the tRF spectrum of atopic dermatitis (AD) and suggested that plasma exosome tRF-28-QSZ34KRQ590K may be a potential biomarker for pediatric patients with AD 94. The expression levels of tRF-16-79MP9PD and tRF-28-OB1690PQR304 in the plasma of the children with obstructive sleep apnea-hypopnea syndrome (OSAHS) are significantly decreased, and may become new biomarkers for OSAHS diagnosis 95. In patients with diabetes nephropathy, the expressions of tRF-5GluCTC, tRF-5AlaCGC, and tRF-5ValCAC are significantly upregulated, while the expressions of tRF-5GlyCCC, tRF-3GlyGCC, and tRF-3IleAAT are significantly downregulated 96. tRF-36-F900BY4D84KRIME, tRF-23-87R8WP9IY, and tRF-40-86J8WPMN1E8Y7Z2R may play a crucial role in the occurrence and development of varicose veins 97.

## 4. Conclusion and Perspective

Although tRFs and tiRNAs have been paid attention to, it is the tip of the “iceberg”. Currently, tRNA sequencing is considered more challenging than mRNA sequencing. Single-cell tRNA-seq may reveal unique expression and/or modification patterns that reflect the regulatory mechanisms of controlling individual cell protein synthesis 98. Clarifying the mechanism by which tsRNAs in single cells contribute to human diseases will provide profound insights into disease therapies.

Although traditional miRNA sequencing is limited by the highly modified and complex structure of tRNAs, methodological innovations in recent years have significantly improved this situation. (1) AlkB-facilitated RNA methylation sequencing (ARM-seq), which used AlkB demethylase to remove m1A and m3C modifications, exerts efficient and quantitative sequencing to whole transcriptome tRNA, and solves the problem of reverse transcriptase truncation at modification sites 99. (2) PANDORA-sequencing (PANDORA-seq) combines AlkB processing and T4 polynucleotide kinase (T4PNK)-mediated end repair to efficiently identify and sequence tsRNA with 2',3'-cyclic phosphate (2',3'-cP) termini 100. This technique reveals large amounts of tsRNA and rsRNA that are previously missed by traditional methods and identifies key regulatory molecules in reproductive biology and cancer. (3) Although PANDORA-seq integrated repair of cP termini, earlier studies and specific optimization protocols (sometimes referred to as cP-seq strategies) had emphasized the importance of specific enrichment of tRNA halves produced by Angiogenin cleavage 101. These methods convert unligable cP termini into standard linker ligation substrates by enzymatic conversion, and are key to resolving tsRNA dynamics in stress response. (4) In addition, YAMAT-sequence 102 provides an efficient alternative to detect tRNAs by optimizing linker ligation conditions without enzyme pretreatment. Meanwhile, sequencing methods capable of single-base resolution localization modification (including MLC-sequencing, other cutting-edge attempts, and validation strategies combined with mass spectrometry) are constantly being developed to further explore the specific impact of modification on tsRNA function. These technological advances suggest that tRNA/tsRNA sequencing is no longer an “insurmountable” challenge, but a new era of precision and panorama. Ignoring these advances would not accurately reflect the current state of scientific research.

Furthermore, to clarify the structural characteristics of tsRNAs, future studies should focus on elucidating the functions of their components. However, methodologies for identifying tsRNAs remain underdeveloped, necessitating the establishment of effective research approaches to systematically investigate tsRNAs structures and their mechanisms of action. Accumulating evidence suggests that tRFs are involved in multiple aspects of cancer and may serve as potential biomarkers for cancer diagnosis and prognosis. Currently, tsRNA nomenclature, such as tsRNA, tiRNA, sitRNA, semi tRNA, is still irregular. Many studies believe that tsRNA is a more appropriate name. Different sequencing methods yield different results, resulting in significant heterogeneity. Consistent quality control standards and standardized operations can reduce differences at certain extent. tsRNAs are unstable and easily degraded, which makes their detection difficulty. However, there are practical challenges to their clinical application. It needs to develop novel methods to overcome RNA degradation. In summary, as technology advances, an increasing number of tsRNAs will be identified and characterized in the future, and understanding their biological roles and clarifying their regulatory mechanisms is the most important and difficult task.

## Figures and Tables

**Figure 1:**
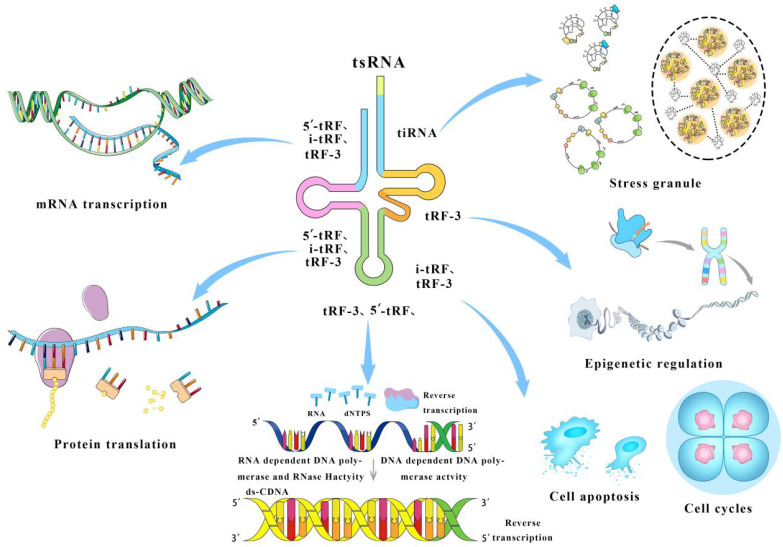
Function and mechanism Schematic of tsRNAs participating in various biological activities. 5'-tRF, i-tRF, and tRF-3 mediate the inhibition of mRNA transcription. 5'-tRF, i-tRF, and tRF-3 inhibit protein translation. tRF-3 and 5'-tRFs regulate reverse transcription through interaction with reverse transcriptase. i-tRF and tRF-3 regulate cell cycle process and participate in cell apoptosis. tRF-3 participates in epigenetic inheritance. tiRNAs induce stress granules.

**Table 1:** tsRNAs from patients with various tumors

Disease	Sample types	tsRNA	Reference
Pancreatic cancer	Serum	tRFProAGG004,tRFLeuCAG002	81
Pancreatic ductal adenocarcinoma	Plasma, Tissue	tsRNAValTAC41、tsRNAMetCAT37	82
Chronic lymphocytic leukemia	CD5+/CD19+B cell	tRNAHis	83
Chronic lymphocytic leukemia, lung cancer	Tissue	tsRNA-46 and tsRNA-47	84
Ovarian cancer	Tissue	tsRNA-29	84
Hepatocellular carcinoma	Serum	tRFGlnTTG006	85
Liver cancer	Plasma exosomal	tRNA-5	86
Gastric cancer‌	Plasma	tRF-33-P4R8YP9LON4VDP	87
Lung adenocarcinoma	Plasma, Tissue	tRF-16-L85J3KE,tRF-21-RK9P4P9L0,tRF-16-PSQP4PE	88
Muscle-invasive bladder cancer	Tissue	tiRNA-1:33GlyGCC1,tRF-1:32GlyGCC1,tRF-+1:T20-SerTGA1	89
Glioblastoma, Low-grade glioma	Tissue	tRF-1- 32-chrM	90
Laryngeal squamous cell carcinoma	Tissue	tRFTyr	91
Non-small cell lung cancer	Plasma exosomal	tRFLeuTAA005,tRFAsnGTT010,tRFAlaAGC036,tRFLysCTT049,tRFTrpCCA057	92
Breast cancer	Plasma, Tissue	tRFGluCTC003,tRFGlyCCC007,tRFGlyCCC008, tRFLeuCAA003,tRFSerTGA001,tRFSerTGA002	93
Multiple myeloma	Bone marrow‌	tiRNA-1:34GluTTC-2,tRF-60:76ArgACG1M2	94
Colorectal cancer (CRC)	Plasma exosomal, Tissue	tRF-3022b,tRF-3030b,tRF-5008b	95

**Table 2:** tsRNA applications in therapeutic and prognosis of tumor patients

Disease	Sample type	tsRNA	Reference
Pancreatic cancer	Tissue	tRFProAGG004,tRFLeuCAG002	81
Colorectal cancer	Tissue	tsRNA-40	84
Colorectal cancer	Tissue	i-tRFGlyGCC	96
Lung adenocarcinoma	Plasma, Tissue	tRF-21-RKP4P9L0	88
Breast cancer	Serum	tRF-16-K8J7K1B	97

**Table 3:** tsRNAs from non-tumor diseases

Disease	Sample types	tsRNAs	Reference
Systemic lupus erythematosus (SLE)	Serum	tRFHisGTG1	74
Hormone Osteonecrosis of Femoral Head (SONFH)	Plasma exosome	tsRNA-10277	75
Pulmonary arterial hypertension (PAH)	Plasma	i-tRF-31:54ValCAC1,5'tiRNA-31GluCTC16,tRF-3aAspGTC9,tRF-3bTyrGTA4,5'tiRNA-33LysTTT4,i-tRF-8:32ValAAC2,i-tRF-2:30HisGTG-1, i-tRF-15:31LysCTT1	37
Osteoporosis	Plasma exosome	tRF-25, tRF-38, tRF-18	76
Atopic dermatitis (AD)	Plasma exosome	tRF-28-QSZ34KRQ590K	77
Obstructive sleep apnea-hypopnea syndrome (OSAHS)	Plasma	tRF-16-79MP9PD, tRF-28-OB1690PQR304	78
Diabetic nephropathy (DN)	Serum	tRF-5GluCTC, tRF-5AlaCGC,tRF-5ValCAC,tRF-5GlyCCC,tRF-3GlyGCC,tRF-3IleAAT	79
Varicose veins (VVs)	Vascular tissue	tRF-36-F900BY4D84KRIME,tRF-23-87R8WP9IY, tRF-40-86J8WPMN1E8Y7Z2R	80
